# Severe infantile epileptic encephalopathy due to mutations in *PLCB1*: expansion of the genotypic and phenotypic disease spectrum

**DOI:** 10.1111/dmcn.12450

**Published:** 2014-03-29

**Authors:** Adeline Ngoh, Amy McTague, Ingrid M Wentzensen, Esther Meyer, Carolyn Applegate, Eric H Kossoff, Denise A Batista, Tao Wang, Manju A Kurian

**Affiliations:** 1 Neurosciences Unit, Developmental Neurosciences, University College London, Institute of Child HealthLondon, UK; 2 Department of Neurology, Great Ormond Street HospitalLondon, UK; 3 McKusick-Nathans Institute of Genetic Medicine, Johns Hopkins University School of MedicineBaltimore, MD, USA; 4 Department of Pediatrics, Johns Hopkins University School of MedicineBaltimore, MD, USA; 5 Department of Neurology, Johns Hopkins University School of MedicineBaltimore, MD, USA; 6 Department of Pathology, Johns Hopkins University School of MedicineBaltimore, MD, USA; 7 Kennedy Krieger InstituteBaltimore, MD, USA

## Abstract

Homozygous deletions of chromosome 20p12.3, disrupting the promoter region and first three coding exons of the phospholipase C *β*1 gene (*PLCB1*), have previously been described in two reports of early infantile epileptic encephalopathy (EIEE). Both children were born to consanguineous parents, one presented with infantile spasms, the other with migrating partial seizures of infancy. We describe an infant presenting with severe intractable epilepsy (without a specific EIEE electroclinical syndrome diagnosis) and neurodevelopmental delay associated with compound heterozygous mutations in *PLCB1*. A case note review and molecular genetic investigations were performed for a child, approximately 10 months of age, admitted to Johns Hopkins University Hospital for developmental delay and new-onset seizures. The patient presented at 6 months of age with developmental delay, followed by the onset of intractable, focal, and generalized seizures associated with developmental regression from 10 months of age. Presently, at 2 years of age, the child has severe motor and cognitive delays. Diagnostic microarray revealed a heterozygous 476kb deletion of 20p12.3 (encompassing *PLCB1*), which was also detected in the mother. The genomic breakpoints for the heterozygous deletion were determined. In order to investigate the presence of a second *PLCB1* mutation, direct Sanger sequencing of the coding region and flanking intronic regions was undertaken, revealing a novel heterozygous intron 1 splice site variant (c.99+1G>A) in both the index individual and the father. Advances in molecular genetic testing have greatly improved diagnostic rates in EIEE, and this report further confirms the important role of microarray investigation in this group of disorders. *PLCB1-*EIEE is now reported in a number of different EIEE phenotypes and our report provides further evidence for phenotypic pleiotropy encountered in early infantile epilepsy syndromes.

## What this paper adds

1. It contributes to awareness of the different electroclinical phenotypes of *PLCB1*-related EIEE, and provides further evidence of phenotypic pleiotropy and genetic heterogeneity in EIEE.
2. It emphasises the importance of microarray studies as an early investigation in EIEE.

## 

An epileptic encephalopathy is defined as a clinical syndrome associated with features of encephalopathy that present or worsen after the onset of epilepsy;^1^ although it may present at any age, it is most common in infancy and early childhood.^1^,^2^ The early infantile epileptic encephalopathies (EIEEs) are characterized by frequent pharmacoresistant seizures, abnormalities on electroencephalography (EEG), and neurodevelopmental delay or regression.^2^ Affected individuals are at risk of profound cognitive and motor impairment in the long term. EIEE can cause considerable hardship for families and the identification of the underlying aetiology for EIEE continues to present a significant clinical challenge. The aetiology of EIEE is heterogeneous, and individuals with EIEE are often found to have acquired causes (e.g. hypoxic–ischaemic encephalopathy), structural defects (e.g. cortical brain malformations), or metabolic disorders.^3^,^4^ Over the last decade, rapid developments in molecular genetic techniques have resulted in a huge increase in the number of reports on novel genetic causes of EIEE.^5^,^6^ Genetic analysis for copy number variants^7^,^8^ and single EIEE candidate genes have, therefore, become increasingly important investigations in clinical practice. Recently, homozygous deletions of chromosome 20p12.3, disrupting the promoter region and first three coding exons of *PLCB1*, have been described in individuals with EIEE.^9^,^10^ In this report, we describe a further case of *PLCB1-*EIEE, thereby expanding the genotypic and phenotypic disease spectrum of this genetic form of infantile epilepsy.

## Method

### Clinical case acquisition

The reported patient was evaluated at Johns Hopkins University Hospital, Baltimore, USA at approximately 10 months of age for developmental delay and new-onset seizures. This report has been published with consent from this patient’s parents.

### Molecular genetic investigations

Please see Data S1 (online supporting information) for the molecular genetic investigation methods.^9^

## Results

### Clinical case summary

This female infant was born at 37 weeks’ gestation, after a typical pregnancy and delivery, with a birthweight of 3230g (25th–50th centile) and head circumference of 35cm (25th–50th centile). She is of African-American descent, the second child of healthy non-consanguineous parents, and has no significant family history. From 6 months of age, before the onset of seizures, there was some concern regarding generalized hypotonia and global neurodevelopmental delay. By 10 months of age, she developed catastrophic onset of seizures characterized by bilateral upper limb jerking, associated with eye deviation and staring. The seizures occurred multiple times daily. Some seizures were prolonged, necessitating admission, on one occasion, to the intensive care unit.

On examination at presentation, she had typical growth parameters and her head circumference was on the 75th centile. There were no dysmorphic features. She was noted to have right esotropia with right rotatory nystagmus. There was no iris coloboma or corneal clouding. Axial hypotonia was evident; however, she had good muscle bulk and typical deep tendon reflexes. Examination of the cardiovascular, respiratory, and abdominal systems was unremarkable. Brain magnetic resonance imaging at 11 months of age revealed mild global reduction of supratentorial cerebral volume and a mildly hypoplastic corpus callosum. The image was otherwise normal, with age-appropriate myelination and neuronal migration. Electroencephalography (EEG) at the age of 10 months showed a disorganized background, with diffuse slowing and multifocal spikes throughout the recording (Fig. S1, online supporting information). Occasional 10-second right and left temporal seizures were also noted. Clinical and EEG features were not compatible with any known age-dependent EIEE electroclinical syndrome and therefore can be classified as non-specific EIEE. Extensive laboratory investigations (including blood gas, plasma lactate, ammonia, creatine kinase, carnitine profile, very long-chain fatty acids, transferrin isoelectric focusing, urinary organic acids, guanidinoacetate, and sulphite, and cerebrospinal fluid lactate, amino acids and glucose) were normal.

Despite trials of multiple antiepileptic drug regimens there was no significant improvement in seizure control. A ketogenic diet was implemented with some modest reduction in seizure frequency. Presently, at 3 years of age, the patient continues to have approximately four or five seizures a day, mainly tonic–clonic, and is maintained on treatment with clobazam and rufinamide. She has axial and peripheral hypotonia and there is evidence of severe global neurodevelopmental delay. She is unable to sit or mobilize independently. She does not reach out to grasp objects but is able to hold small objects occasionally when these are placed in her hand. She is able to track objects visually. She does not vocalize. Owing to an unsafe swallow and risk of aspiration with liquids, a gastrostomy tube was recently inserted for fluid intake.

### Molecular genetic investigations

**Figure 1:**
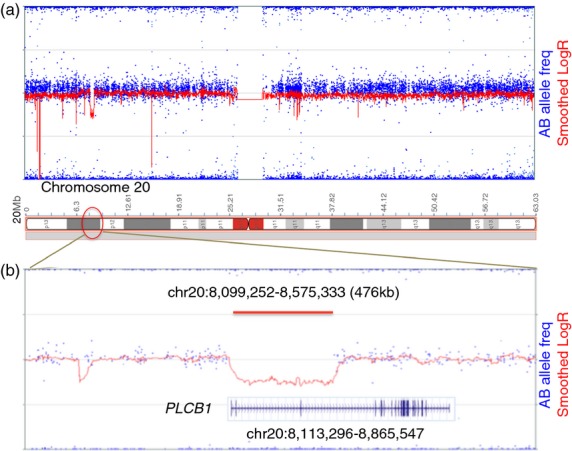
Molecular genetics results data. Panel (a) and (b): Microarray data. Single nucleotide polymorphism array data for chromosome 20 (panel [a]) and the region at 20p12.3 (panel [b]) from the index individual are shown. Relative AB allele frequencies and LogR ratio intensity are indicated. The 476kb deleted region at 20p12.3 in the index individual is highlighted by a red horizontal bar (panels [a] and [b]). The deleted region includes the first three coding exons of *PLCB1* (panel [b]).

**Figure 2:**
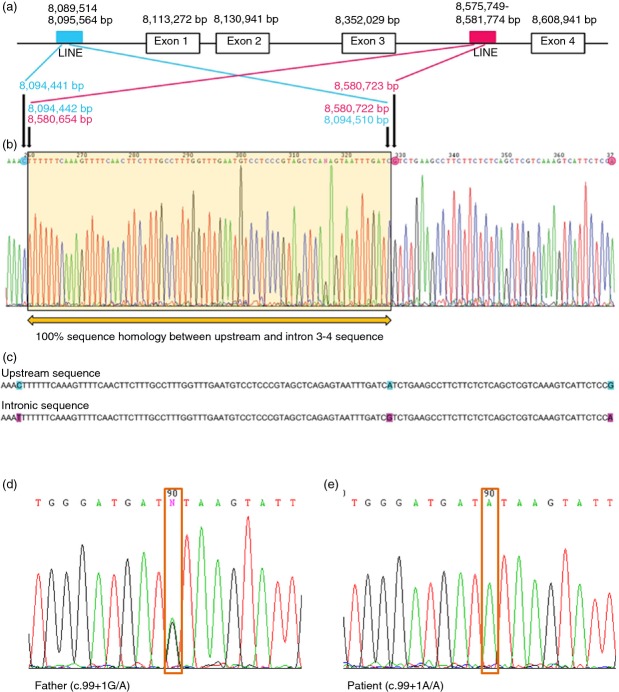
Definition of the genomic breakpoints of the deletion and sequence chromatograms. (a) Schematic diagram of *PLCB1* gene comprising upstream region and exon 1 to 4 with localization of repetitive long interspersed elements. (b) Sequence chromatogram of long range polymerase chain reaction product encompassing breakpoints which are located between 8 094 442 and 8 094 510 bp in upstream sequence and 8 580 654 and 8 580 722 bp in intron 3 sequence. A 68 bp sequence which showed 100% sequence homology for both upstream and intron 3 sequence is highlighted by a yellow box. (c) Comparison of upstream and intron 3 sequence flanking the breakpoint. Differences highlighted by turquoise (upstream sequence) and purple (intronic sequence). (d) Sequence chromatogram from father of index individual. (e) Sequence chromatogram from the index individual. As illustrated, this splice site change appears heterozygous in the father but appears homozygous in the affected child due to absence of the deleted allele.

A routine diagnostic microarray study identified a heterozygous 476kb deletion on chromosome 20p12.3 from base pairs (bp) 8 099 252 to 8 575 333 (Human Genome Build 37; Fig.1). This deleted region, from rs6140539 to rs6118262, encompassed part of the 5′ region and the first three coding exons of *PLCB1*. Microarray studies of parental DNA confirmed the same heterozygous deletion in the mother of the proband and absence of the deletion in the father. The telomeric and centromeric genomic breakpoints were further characterized and mapped to 8 094 442 to 8 094 510 bp and 8 580 654 to 8 580 722 bp respectively (Fig.2). Further analysis revealed that the telomeric and centromeric breakpoints lie within two L1 family long interspersed nuclear elements (LINEs), LIPA3 and LIPA2, occurring at chromosome 20, 8 089 514 to 8 095 564 bp, and chromosome 20, 8 575 749 to 8 581 774 bp. In order to determine whether the proband harboured a second *PLCB1* mutation on the paternally derived allele, direct Sanger sequencing was performed of the whole coding sequence of *PLCB1* (including flanking exon–intron boundaries) for the child and both parents. This revealed a splice site variant in intron 1 (c.99+1G>A) in both the index individual and in the father (Fig.2). This splice site mutation, affecting the first base after exon 1, was predicted to cause deleterious splicing (Berkeley Drosophila Genome project: www.fruitfly.org/seq_tools/splice.html), thereby either leading to nonsense-mediated decay or a truncated protein product.

## Discussion

We report a novel case of *PLCB1-*EIEE (OMIM EIEE12; #613722) with compound heterozygous gene mutations. Our report highlights a new phenotype for *PLCB1*-EIEE as well as a previously unreported point mutation.

*PLCB1* encodes the phosphoinositide-specific enzyme, phospholipase C *β*1. It is activated by G-proteins and catalyzes the generation of second messengers, inositol 1,4,5-triphosphate and diacylglycerol from phosphatidylinositol 4,5-biphosphate, an essential step in the intracellular transduction of a large number of extracellular signals.^11^ PLCB1 plays an important role in modulating diverse developmental and functional aspects of the central nervous system.^12^ In the murine model, biallelic knock-out of *plcb1* results in an epileptic phenotype.^13^ At a cellular level, it is postulated that PLCB1 deficiency selectively impairs muscarinic acetylcholine receptor signalling, thereby disrupting normal inhibitory neuronal circuitry, increasing neuronal excitability, and lowering the seizure threshold.^9^,^13^

Our patient represents the third case of *PLCB1*-EIEE reported in the literature. Notably, our case differs from the previously described cases in both genotype and phenotype. The first reported case from 2010 had catastrophic onset of focal and tonic seizures in early infancy with evolution into West syndrome at 8 months of age.^9^ The second case had a different electroclinical diagnosis of malignant migrating partial seizures in infancy.^10^ Our patient represents a case of non-specific EIEE. Despite these differences, there are a number of common features including (1) seizure onset in infancy; (2) pharmacoresistant seizures; (3) abnormalities on EEG; (4) neurological/developmental regression associated with seizures; and (5) long-term cognitive and motor neurodevelopmental impairment.

Our findings also highlight the extensive phenotypic pleiotropy seen in EIEE, clearly illustrated by other cases of genetic EIEE such as those due to mutations in *STXBP1*,^14^–^17^ *ARX*,^6^,^18^ and *SCN1A*.^19^,^20^ Phenotypic pleiotropy and genetic heterogeneity are increasingly recognized in EIEE. Whilst in general terms, such advances in improved genetic diagnosis are highly beneficial, on another level, it certainly renders genetic investigation of EIEE a diagnostic challenge for the clinician.^21^ It is likely that, in the future, (1) single gene testing will be limited to situations in which the clinical history and phenotype are highly suggestive of a specific gene; and (2) targeted massively parallel resequencing/multiple gene panels for candidate EIEE genes (including *PLCB1)* will play an increasingly important role.^22^

Our report further emphasizes the importance of diagnostic microarray analysis in EIEE.^8^,^21^ Detection of the heterozygous deletion on chromosome 20p12.3 prompted further genetic investigation of *PLCB1* and the identification of the second point mutation, thereby leading to the diagnosis of *PLCB1*-EIEE. Rare copy number variants are increasingly recognized as an important cause of EIEE, and chromosomal microarray analysis should be carried out as an early investigation in the diagnostic work-up of such cases.^7^,^21^ Whilst both of the cases of *PLCB1*-EIEE previously reported were associated with a homozygous *PLCB1* deletion,^9^,^10^ our patient was found to harbour a heterozygous deletion in tandem with an intron 1 splice site variant.

Interestingly, the deletion detected in this patient has almost identical breakpoints to those described in the individual with West Syndrome *PLCB1*-EIEE.^9^ It is likely that the LINEs (which represent highly homologous repetitive sequences) at the breakpoint sites predispose to chromosomal rearrangements, thereby playing an important mechanistic role in the way in which recurrent deletions occur. The second point mutation described in our individual is a novel splice site variant affecting the first base after exon 1 of *PLCB1*. To date, no point mutations have yet been described in *PLCB1-*EIEE and our report, therefore, provides further insight into other disease causing mutations within the spectrum of *PLCB1-*EIEE. Identification of both of the disease-causing mutations in our patient allowed us to confirm a genetic cause for this patient’s EIEE. Establishing a genetic aetiology for an individual’s epilepsy affords a number of benefits. It provides families with accurate information about risk of recurrence and thereby facilitates prepregnancy counselling, prenatal diagnosis, and preimplantation diagnosis. Confirmation of a genetic diagnosis can also help limit more invasive diagnostic investigations (such as lumbar puncture and tissue biopsy). For certain genetic disorders, diagnosis can guide treatment^23^,^24^ and discussions regarding prognosis.^24^,^25^ From a broader perspective, identification of causative genes contributes greatly to the understanding of disease mechanisms in EIEE and it is also likely that it will pave the way for the development of novel targeted drug therapies.

## Conclusion

In conclusion, we report a novel case of *PLCB1-*EIEE in an child presenting with developmental delay and epilepsy. Autosomal recessive *PLCB1*-EIEE is an emerging cause of infantile epileptic encephalopathy, causing a number of different electroclinical phenotypes. It is likely that such genetic characterization of EIEE cases will lead to a better understanding of genotypes and phenotypes, as well as more elucidation of disease mechanisms and therapeutic strategies.

## Abbreviations

EIEE: Early infantile epileptic encephalopathy

## Supporting Information

**Data S1:** Methods: molecular genetics investigations.

**Figure S1:** Electroencephalogram recordings from the proband.
