# Supplementary material for: Severe infantile epileptic encephalopathy due to mutations in PLCB1: expansion of the genotypic and phenotypic disease spectrum
Source: Dev Med Child Neurol. 2014 Mar 29;56(11):1124–8. doi: 10.1111/dmcn.12450 (PMC4230412; doi:10.1111/dmcn.12450)
Supplement: Supplementary file 1 — Data S1: Methods: molecular genetics investigations. [file dmcn0056-1124-sd1.docx]

**Supplementary data**

**Methods: Molecular Genetics Investigations**

Microarray studies: Single nucleotide polymorphism (SNP) array studies were performed at

the Cytogenetics Laboratory of the Kennedy Krieger Institute (Baltimore, MD). An SNP array

was performed for the index case using the Illumina Human OMNI 1M Quad Beadchip with

over 1,000,000 markers, and for the parents with the CytoSNP12 with 300,000 markers

(Illumina, San Diego, CA) following the manufacturer’s protocols. Beadchips were imaged

using Illumina BeadArray reader. Allelic composition and signal intensity were analysed with

the KaryoStudio 1.2 and Genome Studio v1.1.9 softwares and CNVPartition v2.4.4.0

algorithm (Illumina, San Diego, CA).

Characterisation of the putative *PLCB1* deletion: To determine the *PLCB1* deletion

breakpoints, long-range polymerase chain reaction (PCR) techniques were employed using

the PCR extender system (Flowgen Bioscience, Nottingham, UK) as already described.9

Previously designed sequential forward primers were utilised for sequencing the deletion

specific-amplicon in order to determine the deletion breakpoints.

Sanger sequencing of *PLCB1* for genomic and complementary DNA (cDNA): The DNA

template of *PLCB1* was taken from Ensembl genome browser

(http://www.ensembl.org/index.html), chromosome position 20p12.3, NC_000020.10

(8,112,824–8,949,003bp). Based on all Ensembl coding transcript variants of *PLCB1*, primer

pairs for exon-specific PCR amplification of the genomic exons (and flanking exon-intron

boundaries) were utilised.^9^ Previously designed primer pairs for amplification of cDNA

fragments of *PLCB1* transcripts were also utilised. PCR amplification of genomic DNA and

cDNA was performed according to standard protocols with BioMix™ Red (Bioline Ltd.,

London, UK). The PCR products were purified with MicroCLEAN (Web Scientific, Crewe, UK)

or gel-extracted (QIAGEN, Manchester, UK), then directly sequenced by Big Dye Terminator

Cycle Sequencing System (Applied Biosystems, Foster City, USA). The EDTA method of

precipitation was utilized for post sequencing clean-up. Sequencing reactions were run on an

ABI PRISM 3730 DNA Analyzer (Applied Biosystems) and then analyzed using Chromas

software (<http://www.technelysium.com.au/chromas.html>)
