# Supplementary figures and images for: Severe infantile epileptic encephalopathy due to mutations in PLCB1: expansion of the genotypic and phenotypic disease spectrum
Source: Dev Med Child Neurol. 2014 Mar 29;56(11):1124–8. doi: 10.1111/dmcn.12450 (PMC4230412; doi:10.1111/dmcn.12450)

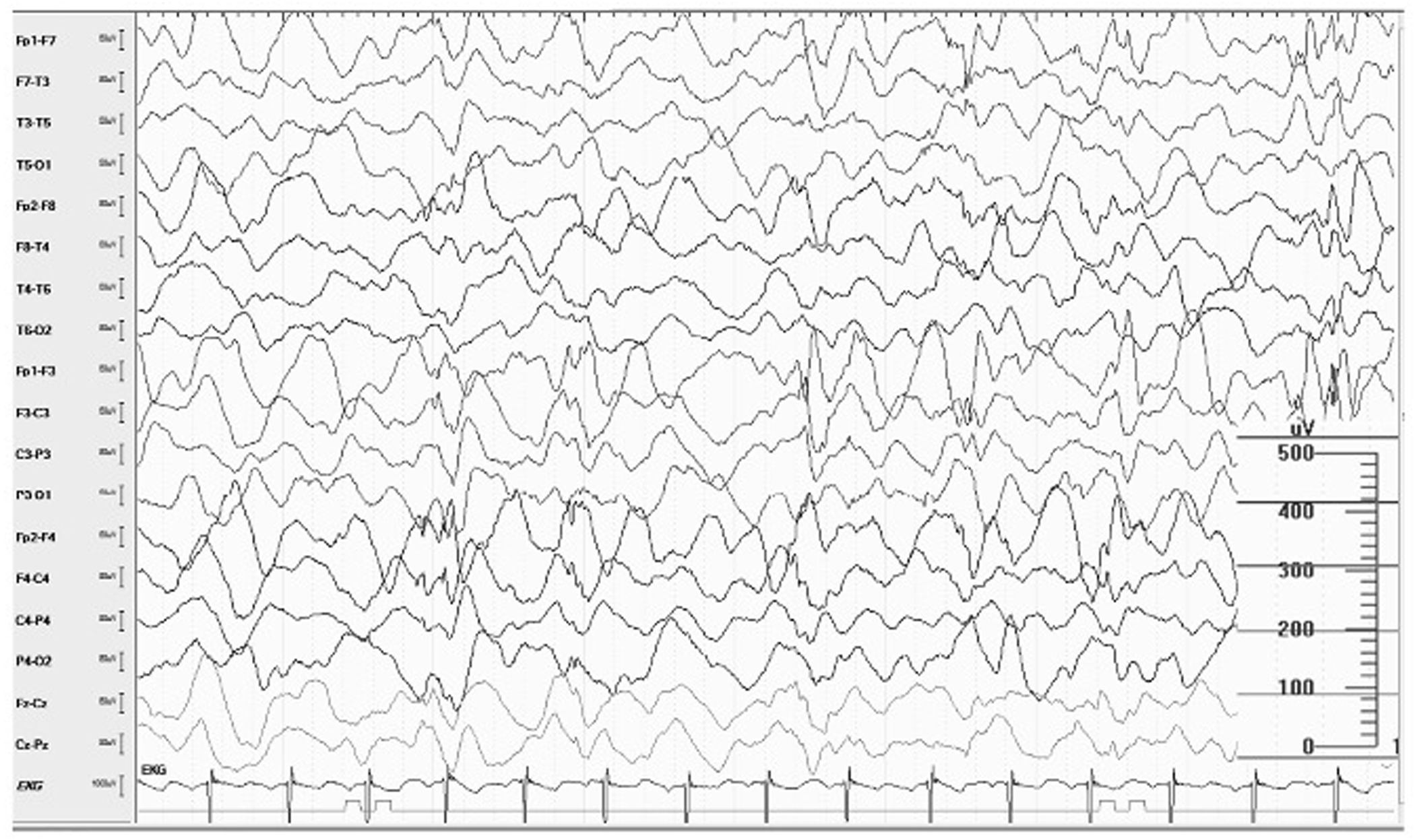

Supplement: Supplementary file 2 — Figure S1: Electroencephalogram recordings from the proband. [file dmcn0056-1124-sd2.tif]
