# Supplementary material for: Severe infantile epileptic encephalopathy due to mutations in PLCB1: expansion of the genotypic and phenotypic disease spectrum
Source: Dev Med Child Neurol. 2014 Mar 29;56(11):1124–8. doi: 10.1111/dmcn.12450 (PMC4230412; doi:10.1111/dmcn.12450)
Supplement: Supplementary file 3 [file dmcn0056-1124-sd3.docx]

**Supplementary Figure Legend:-**

**Supplementary Figure: Electroencephalogram recordings from proband**

EEG at 10 months of age. Disorganized pattern with diffuse slowing and high voltage,

multifocal spikes. The amplitude of spikes and slow wave activity is not high enough to meet the criteria for hypsarrhythmia. Unlike typical hypsarrhythmia, there is some synchrony between the hemispheres.
